# Exploring Ageism as a Structure of Consciousness Across the Female Life Course Through the Work of Simone de Beauvoir

**DOI:** 10.1093/geront/gnac123

**Published:** 2022-08-15

**Authors:** Susan Pickard

**Affiliations:** Department of Sociology, Social Policy and Criminology, University of Liverpool, Liverpool L69 7ZA, UK

**Keywords:** Ageism, Double standard of aging, Othering of old age, Sexism, Simone de Beauvoir

## Abstract

Drawing on the work of Simone de Beauvoir, this paper considers ageism as a structure of consciousness, with important convergences with, as well as departures from, those associated with gender. Specifically, it explores the double consciousness through which the self is experienced negatively as old, at 2 points in a woman’s life course, namely midlife and “old age.” The first kind of ageism works through the double standard of aging and the gender hierarchy, whereby women can no longer successfully comply with the objectification of the male gaze. This is a moment of intense age consciousness, tainted by a gendered narrative of decline, working jointly from the outside in and from the inside out. The second form of ageism has its source in the social world, in the reactions of other people to old age, resulting in an uneasy dialectic between one’s own sense of an ageless or youthful self and the alien view of self as old. The paper proceeds as follows. After a brief account of Beauvoir’s general philosophical approach, I compare and contrast the doubled consciousness associated with aging as a woman and with aging in a more general sense as described in *The Second Sex* and *The Coming of Age*, respectively. I then flesh out this conceptual framework by drawing on fiction and autofiction written by Beauvoir herself. Together, this approach vividly illuminates the important interconnections and intersections between ageism and sexism, the way they work together, as well as separately, across the female life course.

In this paper I revisit the concept of ageism through the work of Simone de Beauvoir. Focusing on the interplay between social structure and lived experience as they give rise to structures of consciousness, I look at two forms of “double consciousness” associated with ageism. The first, connected with the double standard of aging, arises out of the gender system that configures the feminine as simultaneously subject and object. The second is another, contrasting, form of double consciousness that is generated by the Othering of old age. Both forms of double consciousness are features of oppression and in this paper I will draw out the similarities, contrasts, and points of intersection between ageism and sexism as forms of oppression, as they feature, respectively, in two of Beauvoir’s major works, namely *The Second Sex* and *The Coming of Age*. Both texts share a similar structure describing the oppression of women and older people, respectively, as a result of interaction between two axes: that of social structure and that of individual experience. Bringing age and gender together in this way in a consideration of ageism addresses a lacuna noted by Clary Krekula when she writes: “Within the gender theoretical sphere, older women have tended to be left out, and age and ageing are seldom addressed,” whereas at the same time “social gerontology lacks a widespread connection to theoretical advances in gender research” (2007, pp. 155–156).

Using the lens of Beauvoir’s life-course theory of “becoming woman” also enables us to explore the “age” part of “ageism” and to view it as consisting of strands that work in different ways at different points in the life course. Thus, for instance, the double standard of aging is a form of ageism that affects women far earlier than men, “aging” women earlier and more powerfully, and deeply shaping their experience of midlife. Ageism in deep old age works rather differently and although it shares elements of this gendered ageism, and in lived experience they can and do overlap, the two can also be distinguished, certainly conceptually and to some extent experientially.

In what follows, I will first set the scene by examining Beauvoir’s general theoretical position and then I will delineate these two forms of double consciousness in more detail. Turning then to lived experience, I will draw on Beauvoir’s literary works, specifically her fiction. To do so is in keeping with Beauvoir’s own aims and practices as a philosopher. Her commitment to literature as a means of expressing and expanding on her philosophical ideas was formulated when she was a student and indeed all her life she remained frustrated by the dry and dusty abstractness of much philosophy of the day. Writing fiction and autofiction allowed her to approach the stuff of everyday, particularly women’s, lives which were not normally within the purview of philosophers, and to do so via a phenomenological narrative method (Kirkpatrick, 2019).

## Theoretical Prologue: Simone de Beauvoir’s Body-as-Situation

For Beauvoir, the experience of embodied events—such as menstruation, menopause, or aging—derives from cultural norms and attitudes, pregiven meanings which are handed down and which we absorb both tacitly and explicitly. A woman’s body will be experienced differently in societies where she is not valued for her youth and fertility, for example, but rather for her experience and leadership (for example), or ones in which the male body has not classically been taken as a standard in medicine and biology. Beauvoir writes: “viewed in the perspective that I am adopting—that of Heidegger, Sartre, Merleau-Ponty—the body is not a thing, it is a situation: it is our grasp on the world and a sketch of our project” (1997, p. 46). Thus, while women are also free, there are limits to this freedom which itself is part of their “situation.” Similarly in old age a mixture of social attitudes and biological changes (which in turn shape how these changes are interpreted) limit freedom to pursue one’s projects. Sartre argued that a temporary loss of freedom occurs ordinarily in life when I am rendered “object” in relation to another, turned into a “being-for-others.” Beauvoir’s analysis first of women’s situation, and later of older people’s, departing from Sartre’s, suggests rather that for both groups this is not temporary, but more enduring, an inherent feature of social life, owing to the patriarchal and ageist structures of society (Deutscher, 1997; Kruks, 2013). The body-as-situation is a keystone of Beauvoir’s method (alongside other phenomenologists such as Merleau-Ponty), serves “to avoid dividing lived experience up in the traditional subject/object opposition” (Moi, 2005, p. 65) and is used by Beauvoir to “maintain, rather than resolve, the tension between freedom and determinism in human existence” (Mann, 2017, pp. 41/42). Moreover, within this framework, as one’s body provides our grip on the world, consciousness is body-specific and gendered. That is, in men, as masculinity is compatible with action and assertion, there is no friction between body and subjectivity. For women, this is not the case, as I explain in the next section.

I turn next to Beauvoir’s theories of the double consciousness that underpin ageism. In order to set out women’s situation as they age, I will need to take a life course perspective and I thus begin with the kind of consciousness that is crucial to the youthful experience of becoming a woman. I will show that this particular form of doubled consciousness is also germane to the experience of aging for women.

## The Special Double Consciousness Associated With Becoming a Woman

The line for which Beauvoir is most famous is that “One is not born but becomes a woman” (1997, p. 295). *The Second Sex*, her most well-known work, sets out to explicate what she means by this, over the course of two volumes, and some 700 pages. For the purposes of this paper, I will focus on the emergence of the special double consciousness that is intrinsic to becoming a woman. Indeed, one both becomes a woman, and leaves womanhood behind, within the framework of the male gaze. Beauvoir holds that one becomes a woman among other things by making oneself “prey” to the male gaze and male sexual attention. Upon the time of puberty, she explains, when a girl first becomes “visible” to the male gaze, the female child’s subjectivity, which up to that point had scarcely been conscious of itself, becomes doubled, meaning that she sees her own body/self as at once the locus of her subjectivity (as it was before puberty) and simultaneously as an object reflected in the male gaze. While this lies at the heart of her erotic experience it sparks a friction that cannot easily be resolved while she is sexually active. Unlike other kinds of doubled consciousness, as noted it is about *making oneself* an object within the male gaze (*se faire objet* in the original French), meaning that complicity is a key aspect of women’s subjugation. Philosopher Jennifer McWeeny clarifies: “Her body is both the locus of her subjectivity and the instrument for another’s desires; it is jointly that which she lives as her own and that which she lives as if it were someone else’s” (2016, p. 159).

Women’s specific form of double consciousness—the making oneself an object—shares both similarities and differences with other forms of doubled consciousness in ways whose importance will become apparent when we discuss aging and old age. For example, both W. E. B. du Bois and Frantz Fanon wrote about the double consciousness arising from racism in White dominant society. du Bois wrote: “One ever feels his two-ness,—an American, a Negro; two souls, two thoughts, two unrecognized strivings; two warring ideals in one dark body whose dogged strength alone keeps it from being torn asunder” (1897, p. 194). Fanon also describes this experience vividly noting how the Black man is “battered down” by White narratives of blackness including stereotypical associations with “tom-toms, cannibalism, intellectual deficiency, fetishism, racial defects, slave ships …” (1986, pp. 84–85). Other Black American writers, such as James Weldon Johnson and Richard Wright also used and adapted the concept of double consciousness. For example, James Weldon Johnson (1927) rechristened it “dual personality” and applied it to a light-skinned man who can “pass” and who is somehow both Black and White. Richard Wright (1964) referred to it as “double vision” rather than double consciousness; he suggested the concept of a “frog perspective” which always involved some feeling of “looking from below upward” (Gilroy, 1993, p. 161).

McWeeny (2016) suggests that the difference between double consciousness in the forms described above and the variant specific to femininity which involves an aspect of willingness is that the former double consciousness comes from the outside, as a result of inscriptions whereas “*se faire objet* is a consciousness that doubles itself” (p. 160). This coconstitution by women themselves of their doubled self means that it is very difficult for women to evade or end: “The hallmark of a woman’s consciousness is the inescapability of this ambiguity; try as she might, a woman will never succeed in embracing one of the perspectives present in her consciousness to the exclusion of the other” (p. 160). Racism, she notes, usually takes place without this kind of complicity and is thus more likely to give rise to resistance.

The difference can also be captured through the concept of the Master-Slave dynamic of Hegel, which both Fanon and Beauvoir employed to describe the situation of Black men in White society and women in patriarchal society, respectively. Fanon describes how the White man willingly “recognizes” the Black man (thereby, however, denying the Black man’s need to demand such recognition from the White man, as he complained). In contrast, Beauvoir suggests that woman is not able to enter the Hegelian dialectic and achieve recognition at all. As Direk explains: “A relation between the same and the Other who is irreducible to the same can never be a relation of equality and is likely to continue being a relationship of domination, which is different than the slavery of the similar other to the same” (Direk, 2011, p. 60).

## The Double Standard of Aging

The paradox and ambiguity that lie at the heart of femininity—as simultaneously subject and object—generates a long-standing tension that endures throughout a woman’s life, in that both freeing oneself from this particular kind of double consciousness and acquiescing to it give rise to problems. Beauvoir notes: “It is this conflict that especially marks the situation of the emancipated woman. She refuses to confine herself to her role as female, because she will not accept mutilation; but it would also be a mutilation to repudiate her sex.” (1997, p. 691). It is also the conflict that characterizes the aging woman. This is not a conflict experienced by men, for one simple reason: man’s “sexual life is not in opposition to his existence as a person” (1997, p. 64); in other words, he remains an agent and a subject seamlessly throughout love and work. But the essence of being sexually attractive and desirable, as Susan Sontag (1972) has described in her discussion of the double standard of aging, adheres to the template of the youthful, fertile woman, or the appearance thereof. As she suggests, feminine attractiveness has one standard only, that of the extremely youthful woman, unlike for men, where more mature forms of attractiveness—both that of the “man” and that of the “boy”—are possible. However, Beauvoir suggests that the esthetics that govern the double standard and mark an older woman as undesirable are in fact constructed upon male fear of her powers. She notes: “If she ceases to be an erotic object, it is not only because her flesh no longer has fresh bounties for men; it is also because her past, her experience, made her, willy-illy, a person,” an independence which men find “intimidating” (1997, p. 590).

In later life, Beauvoir notes, women slowly lose the ability to make themselves sexual objects while resultantly and more positively, this particular doubled consciousness can be discarded and a seamless, unified consciousness regained. Many women resist this and rather strive, through cosmetic, hormonal, and other practices and treatments, to maintain their place in the youthful regime for as long as possible. The feeling of oneself as aging is acutely foregrounded in whatever choice is made, however, and ageism here is embedded in the appraising male gaze, through which perspective women also view themselves. With aging, the loss of this self-objectifying form of double consciousness is thus replaced not with a new freedom in the form of a sexual agency but rather, for many, with a feeling that one is “too old” for love and sex (this may be different for older women with female lovers, however, e.g., Segal, 2007).

In other words, for women, the double-edged sword lies in the fact that the freedom from the oppression of the doubled consciousness, embedded within the gender hierarchy and into which they enter on “becoming a woman,” is at the other end replaced by the oppression of age. That is, women go from being objects of the male gaze to being invisible and/or powerfully devalued within it. Here, ageism is built into, and works through, the gender system, a way of oppressing women by limiting their value and capital as women. Yet, to be clear, Beauvoir herself sees this double standard of aging as both an oppression—a loss of sexuality—and an opportunity for women to reclaim their original unified subjectivity.

In the next section, I will look at how this kind of struggle, and the direct experience of gendered ageism, is presented in her novel, *The Mandarins*.

### *The Mandarins* and the Double Standard of Aging in Midlife

*The Mandarins* (1993) follows the character of Parisian, Anne Dubreuilh, a practicing psychiatrist, a wife, and a mother aged 39, on her journey from confident, self-possessed professional woman to a woman in emotional turmoil, and indeed on the brink of suicide. At the start of the novel, she is secure in a sexless but emotionally supportive and intellectually companionable relationship with her husband, Dubreuilh, and has put aside questions of sexuality and femininity. When she meets and begins an affair with a charismatic American novelist (Lewis Brogan), she at once begins to see herself again according to the terms of the male gaze. This sexual reawakening is a source of pleasure but also of exquisite suffering on account of her perception of herself as aging and thus at the constitutive limits of desirable femininity. Gazing around her, she begins to perceive all women of her own age as decaying and on the point of ruin (though she does not see any of her male contemporaries in such a light, including her husband who is 20 years older than she). The affair with Lewis Brogan is, to Anne, her “last” chance and when it ends she faces “the end of love.” By this stage she has forgotten how, at the start of the novel, she placed little importance on her sexuality and had felt even so that her life was pleasingly full. The reawakening of her erotic self reminds her of the particular embodied, sexualized style she had lost and anticipates losing again with the end of the affair. As she sees it, this presents a final opportunity to enjoy her life as a woman. Viewing the women of her own age with a youthful gaze she is sure that: “Old age is awaiting me; there’s no escaping it. Even now I can see its beginnings in the depths of the mirror.” Indeed, this is one of the most potent ways through which the double standard is imposed: through mirrors but especially perhaps through the mirrors of women the same age as oneself. It is how “the Look” works to constrain a woman’s freedom. So, only 39, Anne still possesses a youthful appearance, yet for her, viewed through the narrative of the double standard of aging, ruin is omnipresent: “My face still seems smooth and firm, but overnight the mask will melt, laying bare the rheumy eyes of an old woman” (p. 103). Certainly, her female friends and acquaintances have it worse than her in many cases, especially those who have no careers to rely on or otherwise independent sources of self-esteem. Her friend Paula is in that category; as Paula is replaced by a woman 20 years younger than her in her lover, Henri’s, affections, Anne watches Paula age visibly, a victim of the double standard of aging. Beauvoir emphasizes this by choosing to describe Paula’s appearance through the eyes of Henri. When Paula answers the door: “dumbfounded, he asks himself: ‘What’s happened to her?’ Her hair was put up, revealing the thick nape of her neck; her eyebrows were plucked; she was wearing a suit which was too tight for her” (p. 474). In an attempt to make herself once again an object of her ex-lover’s attention, in the manner of “*se faire objet*,” instead she makes herself ridiculous, “mutton dressed as lamb.” But her sexuality has been at the center of her life and losing this annihilates her sense of value and indeed identity. Anne supports her as she suffers a nervous collapse and considers suicide.

Meanwhile, the resurrection of Anne’s “life as a woman” is equally overwhelming: “my body was rising from the dead” (p. 421), she reflects. In step with such a reawakening comes intense vulnerability, a hypervigilance toward Lewis’s moods, his acts of commission and omission, the way he looks at her, with appreciation or disapprovingly, whether he kisses her with enough feeling, whether there is a trace of indifference. Is she old, or not? She flickers between feeling old and young, old and young, according to his behavior and his gaze, as well as how she feels about her appearance: “No, six months from now, I shan’t have aged much, I’ll see Lewis and he’ll still love me” (p. 665). The next minute, a glimpse of the women who are her contemporaries suffice to remind her of exactly what she is: “They’re old, worn-out hags,” I thought, “and I’m the same age as they” (p. 667). Eventually Anne’s affair with Lewis also comes to an end. While Henri, Dubreuilh, and their male associates are ready to start again, with exciting new plans and bold ventures, for Anne it feels like the end of everything of value is done; she has “outlived … love” (p. 760). The striking thing here is that, although Anne has the career and financial independence that Paula lacks and has never put romantic love at the center of her life, nevertheless the two women share much in common as women: to no longer be part of the sexual regime is to no longer know why one is alive, Anne thinks. This is what Beauvoir means when she describes the freedom brought by later life from this doubled consciousness as important but at the same time an amputation, a mutilation.

These passages show powerfully how the double standard of aging works through the forms of double consciousness peculiar to women, and central to the gender hierarchy, to oppress women at exactly the point in their lives when they might be coming into the height of their powers. What could be maturity and self-assurance collapses instead into a sense of deficit, loss, and constraint. As such Beauvoir’s theories suggest that they emerge first and foremost from women’s position as the “second sex,” not from the oppression of the age system.

In the next section of the paper, I will look at a somewhat different kind of double consciousness, about which Beauvoir writes in *The Coming of Age* (1996) and which I read Beauvoir to suggest is associated with a contrasting social etiology, working not through the male gaze but rather through the “youthful structure of the look” (Woodward, 1991) and impacts on both men and women.

## The Double Consciousness of Old Age

Beauvoir wrote *The Coming of Age* almost 20 years after she finished *The Second Sex*; so perhaps it is not altogether surprising that there is a disjuncture between her depiction of aging in both texts, specifically her omission of women’s lived experience. While describing carefully the inner life of the aging woman in *The Second Sex*, she does not discuss women’s lived experience in her text on aging but presents old age as something set apart from one’s own subjectivity, not as something we feel inwardly but to which one yields through a dialectic understanding that emerges from others’ responses and reactions. As she put it, it is: “A dialectic relationship between my being as he defines it objectively and the awareness of myself that I acquire by means of him” (p. 284). We may initially resist this judgment but ultimately we submit, although it is never characterized by the willingness and collusion that characterizes femininity. Also, unlike the doubled consciousness associated with feminine subjectivity, old age, Beauvoir notes, in *The Coming of Age*, is not experienced in the “for-itself” mode, does not exist for the *older* person herself, which is why an “inner feeling of youth” can remain, for some, for so long (and the converse is also true). This is a peculiarly temporal dimension as Beauvoir notes: “Old age is particularly difficult to assume because we have always regarded it as something alien, a foreign species: ‘Can I have become a different being while I still remain myself?’” (p. 283). This form of double consciousness likewise has similarities and differences to that associated with racism. For example, it lacks the temporal dimension. Similarities include the absolute alienation of this outside view, lacking all complicity which means that the body is viewed from the outside, as an alien object: “Consciousness of the body is solely a negating activity. It is a third-person consciousness” (Fanon, 1986, p. 83). Frailty is one example of a negating label imposed on the aged body from the outside. Empirical research shows that older people find this label or diagnosis catastrophically threatening to their identity and sense of well-being and strongly resist it, exhibiting that warring of two consciousnesses of which Fanon writes. It is well-known within clinical and lay circles that yielding to such a judgment can lead to a downward spiral in health and well-being, even death (Fillit & Butler, 2009).

However, there is certainly a “real” element of bodily experience of aging and some of it is undoubtedly negative involving slowing down, weakening, stiffness, and possibly pain and suffering; this makes it more than simply a meaning for others. Beauvoir notes: “Biological decay brings with it the impossibility of transcendence, of becoming passionately involved; it kills projects …” (1996, p. 443). But at the same time we cannot, even then, extract the aged body from the environment in which ageism, flourishing as a form of oppression, declares that youth is valued and older people are merely “useless mouths” in Beauvoir’s words. Deutscher (2003) describes it thus:

In the experience of being too old to climb or of suffering from menstrual pain, for example, one cannot separate the physiological condition from its mediation by the othering of women and the aged … social inequality produces a body experienced as limiting and in this sense impinges on the ontological freedom (p. 290).

In writing about old age, Beauvoir posits a generic subject using the term “man” and focusing on “gender neutral existents” as inherited from Sartre (Deutscher, 1997). Where she does acknowledge specific differences between the sexes her focus is on class differences in the aging experience, not on any unique situation of women. However, it is certainly possible to distinguish Othering as it relates to (older) women and to old age more generally. Sonia Kruks has described how, in comparison to the “asymmetrical recognition” that characterizes the position of the feminine, the Othering of old age is characterized by “aversion.” She notes: “Old age inspires a biological repugnance; in a kind of self-defence one pushes it far from oneself” (2013, p. 217). Moreover, as true to her method of employing literature to expand her philosophical project, both forms of ageism as they apply to women appear in Beauvoir’s fiction, including the novella *The Age of Discretion* (2006), to which I turn next.

### *The Age of Discretion*: The Ageisms of Looking Back and Looking Ahead

In this long short story, published 3 years before *The Coming of Age*, Beauvoir sets out to discuss the experience of being old as a dialectic, one that fluctuates between two attitudes toward aging. Thus, for the protagonist, positive feelings are juxtaposed with deeply negative views, sometimes in dialogue with her husband and sometimes within herself. The protagonist is an unnamed woman in her 60s, a philosophy teacher and mother to Philippe, wife to André, a scientist. She is retired but remains active in the field and as such “works out” a subjectivity associated with age through such a dialectic, a device, moreover, ideally placed to demonstrate the way the double consciousness of old age works as a vehicle for ageism.

The protagonist begins by highlighting some of the positive elements. First, there is the satisfying feeling of having a long past, accompanying a wealth of achievements and experiences that provide a textured background to the present: “… often, quite unexpectedly, I catch sight of it, a background to the diaphanous present … that gives its colour and its light … Once I used to cherish schemes and promises for the future; now my feelings and my joys are smoothed and softened with the shadowy velvet of time past” (p. 14). But quickly, as various shocks and disappointments occur, the positive subjectivity begins to be joined by more negative aspects. For example, she detects a “sluggishness” and indifference, a lack of vitality in André who tells her that that is a property of youth he will never possess again. Not true, she counters: “I shall never believe that you can no longer create” (p. 42). Indeed, she continues: “Maybe you will make your greatest discovery at seventy” (p. 12). However, although she utters this statement at the time when she is confident that she has, herself, just published her best work yet, when critical reviews begin to appear, her optimism falters and with it comes a reappraisal of aging. Combined with a couple of disagreements with her husband and son, she is soon overcome with gloom. She reflects, mournfully:

I should write no more. Then what should I do? What an emptiness within me—all around me. Useless. The Greeks call their old people hornets. “Useless hornet” Hecuba called herself in The Trojan Women. That was my case. I was shattered. I wondered how people managed to go on living when there was nothing to be hoped for from within (p. 53).

As Chris Gilleard explains, in the dialectic of ageism, the positive and negative threads cannot be conjoined but fluctuate in an unsteady binary, meaning that older people “waver in their identities as aged persons, vacillating between an assertive, intentional ageless ‘I’ and an observed and objectified aged ‘me’” (2021a, p. 101; see also Gilleard, 2021b). However, as well as this dialectic, for women, this oscillation will also include a movement between both forms of ageism, that is, the gendered form associated with the double standard of aging and the form associated with the alterity of old age proper. Indeed, the first seeds of doubt about the reception of her book are planted during a meeting with a younger woman friend, Martine. As the protagonist arrives at the café and sees her friend waiting there for her, wearing a short, colorful dress, she describes her as a “lovely young woman. Forty” (p. 14). We might of course guess from other work by Beauvoir that this woman of 40 does not, so confidently, see herself as young. But in the eyes of a woman of 60, Martine appears to reveal all that the protagonist has lost.

Indeed, this novella richly suggests that the line between the double consciousness of old age and that associated with the gendered double standard of aging is blurred, and hard to draw, at least for a woman in what today we might call the “third age.” After the meeting with Martine she goes home and changes into her “prettiest dress,” in honor of her son’s visit but cannot muster up much enthusiasm:

When I was fifty my clothes always seemed to me either too cheerful or too dreary: now I know what I am allowed and what I am not, and I dress without worrying. Without pleasure either. That very close, almost affectionate relationship I once had with my clothes has vanished (p. 17).

As this particular flavor of ageism comes to the foreground, although she has spoken earlier about tending her body like a “somewhat wanting, old friend who needed my help” (p. 17), she notes how she feels reluctant to bathe with her husband during a trip to the home of Manette, her mother-in-law, in the countryside. She reflects, “I am very unwilling to display myself in a bathing-suit, even in front of Andre. An old man’s body, I said to myself, watching him splash about in the water, is after all less ghastly than an old woman’s” (p. 59).

Indeed, her negative perception of herself as aged is formed, on the one hand in relation to younger women and memories of her younger self, and on the other in relation to her dread of deep old age as represented by her 85-year-old mother-in-law, Manette. Manette appears to be serene and happy and indeed the protagonist herself acknowledges that for Manette old age is the “best time of her life” (p. 59), freed as she now is from the duties and responsibilities she had shouldered for most of her adult life. Yet still, so deep is the alterity of old age for her, that she projects toward her own future with dread:

Age had not taken her powers away, but deep inside her, what went on? Did she think of death? With resignation? With dread? I dared not ask … I sat down opposite her. Whatever happened, if I were to reach eighty I should not be like her. I could not see myself calling my solitude freedom and peacefully drawing all the good from each succeeding moment. As far as I was concerned life was gradually going to take back everything it had given me: it had already begun doing so (p. 61).

She is, thus, projecting all her fears and terrors onto the figure of this serene and fulfilled old woman and is utterly unable to imagine herself in that “situation,” although it is only 20 years in the future. In turn, that ageism directly and powerfully affects how she interprets her own life as a woman in her 60s: “Ahead there were the horrors of death and farewells: it was false teeth, sciatica, infirmity, intellectual barrenness, loneliness in a strange world that we would no longer understand and that would carry on without us” (pp. 70–71). This kind of stereotypical imagery, through which she reads the experiences of her own life and anticipates its future passage, as if through a stencil cut with the patterns and features of decline, is vividly reminiscent of the double consciousness stirred up by racism as depicted in Fanon beaten down by his tom-toms.

However, in the end, for this protagonist looking ahead, the positive feelings win out which is surely a hopeful sign that an authentically age-full subjectivity can be achieved. What is at fault, she works out in dialogue with André, is her habitual view of the life course as one of endless progress and upward movement: that is not authentic, though it is the shape given to the life course by modernity, and thus a current that easily sweeps one along. Also part of this system is the opposite narrative of decline which has two parts for her: that associated with feminine sexuality and that of the transcendent self. However, more modest, but genuine, ambitions, daily joys and relationships, are the way to proceed, the couple agree, keeping the tension between freedom and determinism alive. In this sense, continuity, not Otherness, emerges as an alternative perspective on aging into deep old age.

## Discussion and Concluding Thoughts

A focus on ageism viewed through the lens of Simone de Beauvoir’s theories of gender and age, respectively, has provided an alternative perspective to ageism as it is currently understood within gerontology. This perspective depicts ageism as a structure of consciousness, with at least two different sources and forms, associated with midlife and old age, respectively. Both forms overlap in the lived experience of aging women yet can also be seen as analytically distinct. There is the need to develop this analysis further, mapping the heterogeneous play of the double consciousness across the lived experience of diverse women (and men). At this point it is important to note that Beauvoir has been on the receiving end of much criticism among contemporary feminists for, among other things, being a privileged White woman who has little to say about the lives of less privileged women. However, her aims as a philosopher were to address the general rather than the particular while her phenomenological method could subsequently be applied to others in particular situations (Burke, 2017).

This paper, again only by way of a starting point, permits a different theoretical approach to questions such as: how do the age and gender regimes work separately and together? How do ageism, sexism, and racism compare and contrast as modes of oppression? How best can we weave together insights from gender and age theory to understand the situation of aging women (and men)? In terms of social gerontology, useful avenues for research that build on, and extend beyond, Beauvoir’s insights, could include exploring the impact of the double standard of aging to shape or undermine women’s experience of employment, relationship, health, and embodiment at midlife; and conversely to examine the counternarratives relating to the liberating aspect associated with the end of this form of double consciousness. Later in the life course, research could look at how conditions such as “frailty,” which particularly affects women, may contain strands of both gendered ageism focused on the female body and on the double consciousness that arises from old age more generically.

For Beauvoir, both the existence of the gender hierarchy, and the way we approach old age, represents a way to flee the ambiguity of existence, rather than hold in difficult juxtaposition the truth of human freedom lived in a situation of ineluctable constraint. As Kruks notes, in Beauvoir “oppression functions so as to close down the ambiguities of embodied subjectivity and to deny freedom” (2013, p. 56). Yet, those who “benefit” from this pay an equal and opposite price. For men, advantage is “bought at the cost of men’s flight from the ambiguities of their own embodied existence” (Kruks, 2013, p. 69). For younger people, likewise, it is bought at the price of denying the vulnerability, fra(gil)ilty, and need for care that is a feature of all ages, of embodied life itself, as well as the flux, flow, and impermanence that, in an exquisite paradox, both makes possible, and ultimately limits or even destroys, existential freedom.

## Data Availability

This article does not report data and the data availability policy is not applicable to the article.
